# First Report of *Nocardia asiatica* Presenting as an Anterior Mediastinal Mass in a Patient with Myasthenia Gravis: A Case Report and Review of the Literature

**DOI:** 10.1155/2012/325767

**Published:** 2012-07-16

**Authors:** Rima I. El-Herte, Souha S. Kanj, George F. Araj, Hassan Chami, Walid Gharzuddine

**Affiliations:** ^1^Division of Infectious Diseases, American University of Beirut Medical Center, P.O. Box 11-0236, Riad El Solh, Beirut 1107 2020, Lebanon; ^2^Department of Pathology & Laboratory Medicine, American University of Beirut Medical Center, Beirut 11072020, Lebanon; ^3^Division of Pulmonary and Intensive Care medicine, American University of Beirut Medical Center, Beirut 11072020, Lebanon; ^4^Division of Cardiology, American University of Beirut Medical Center, Beirut 11072020, Lebanon

## Abstract

The spectrum of infections with *Nocardia spp*. is heterogeneous. It has classically been associated with lung, brain, or skin involvement. We describe an unusual presentation of *Nocardia asiatica (N. asiatica)* in an Iraqi patient with myasthenia gravis suffering from a disseminated infection and presenting with an anterior mediastinal cystic mass. *N. asiatica* has only been three times described outside Japan and Thailand, and the rarity of this entity deserves this communication.

## 1. Case 

**Figure 1:**
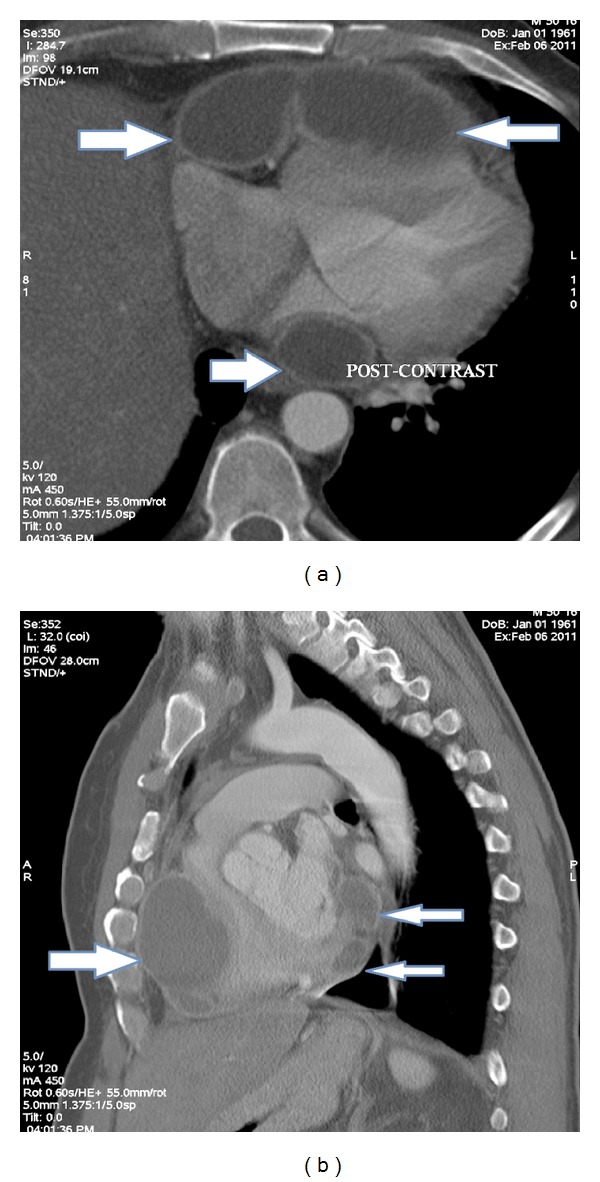
CT scan of chest shows the cysts compressing the right ventricle anteriorly (large arrows) and the cyst behind the heart compressing the left atrium posteriorly (small arrows).

**Figure 2:**
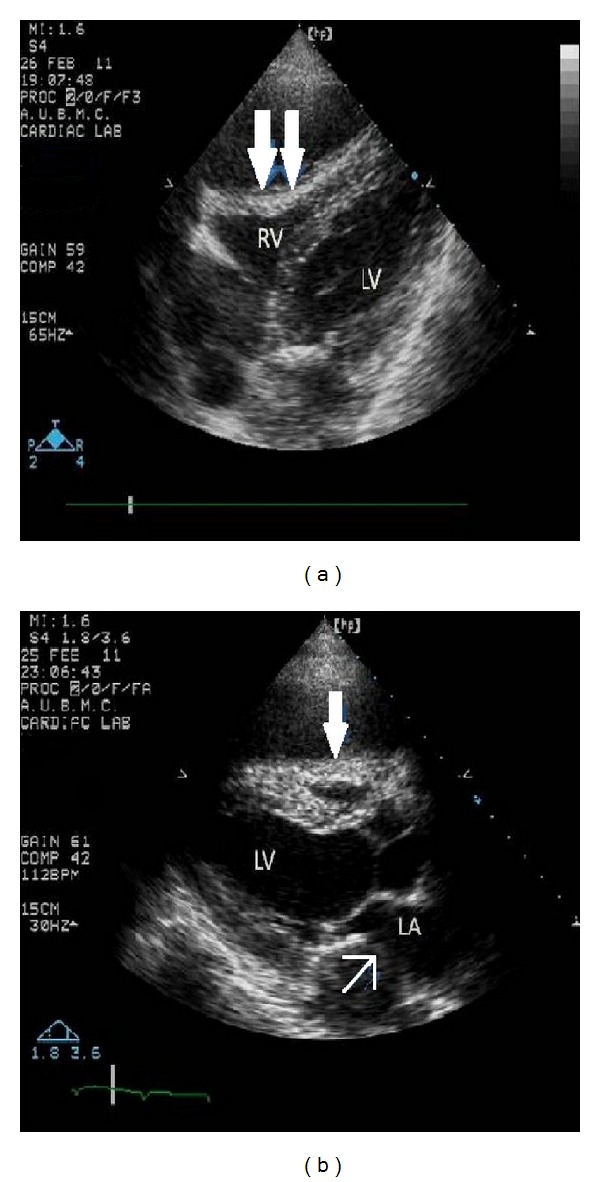
Echocardiography shows the cystic mass surrounding the heart (full arrows) with impingement on the right ventricle and left atrium.

**Figure 3:**
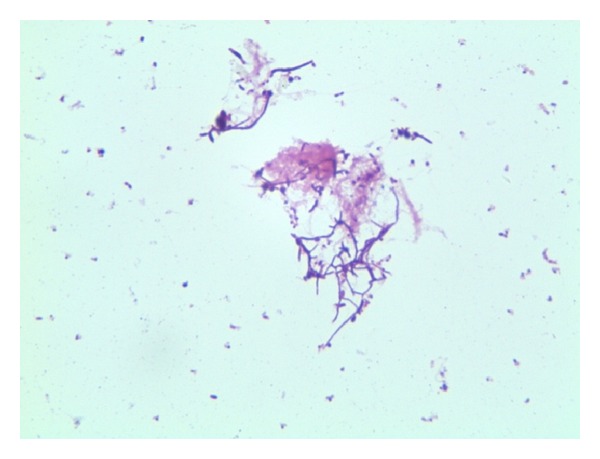
Photomicroscopy of the Gram stain of the aspirated fluid from the cystic masses shows the Gram-positive branching rods indicating the presence of *Nocardia spp. *

A 49-year-old Iraqi male presented in February 2011 to the American University of Beirut Medical Center (AUB-MC) with fever, chills, chest pain, anorexia, and weight loss after being operated on for a presumed malignant thymoma in India. His initial history goes back to May 2010 when he was diagnosed with myasthenia gravis in Iraq and was started on prednisone 60 mg orally and mestinon 60 mg orally three times per day. A computed tomography (CT) of the chest done in Iraq showed a cystic mediastinal mass abutting the right ventricle described as thymoma (images not available) but very similar to the CT done at our institution later. No interventions were undertaken in Iraq. He was then referred for right lateral thoracotomy in India in November 2010. Pathology revealed malignant thymoma. The patient remained hospitalized in India for 3 weeks where he reported chills, weight loss, and sweating without any documented fever. He was started on chemotherapy (unknown regimen) and was discharged home on levofloxacin 500 mg orally daily for ten days. No microbiological work-up was initiated in India to try to detect an underlying infectious process. The period from December 2010 until February 2011 in Iraq was characterized by recurrent chest pain, fever, chills, sweating, cough, and greenish sputum production. Blood tests at that time showed a WBC of 26,000/*μ*L. Repeated CT of the chest showed multiple cystic masses impinging on the right ventricle and left atrium described as metastasis in concordance with his history of malignant thymoma (Figure 1). At a local hospital, he received blood transfusion, fluconazole, and cefotaxime both at unknown frequency and dosage without improvement. He then had a PET scan in Lebanon showing cystic masses in the chest with necrosis and invasion of the pericardium described as metastasis. He presented to our institution where echocardiography showed two large anterior fluid collections one of which was compressing the right ventricle with a third cyst behind the left atrium (Figure 2). Pericardiocentesis was performed and yielded 90 mL of greenish yellow purulent fluid. The Gram stain showed Gram-positive branching rods, and the recovered *Nocardia spp*. was identified in Laboratories Cerba France as *N. asiatica *(Figure 3). The susceptibility testing (Cerba, France) revealed susceptibility to all tested antimicrobials except macrolides, clindamycin, colistin, and aztreonam. A median sternotomy was performed and debridement of the abscesses in the anterior mediastinum as well as biopsy of suspicious tissue was performed. The pathology did not reveal any malignancy. The posterior abscess could not be drained without instituting cardiopulmonary bypass and so was left to be drained with the help of endoscopic ultrasound via the esophagus. 

CT scan of brain showed multiple cystic lesions so the patient was diagnosed as having disseminated *Nocardia asiatica* infection and was started on imipenem, amikacin, and trimethoprim sulfamethoxazole (TMP-SMX) to be given for at least 9–12 months. At five months of followup, the patient is doing well clinically and radiographically.

## 2. Discussion

 In general, infection with *Nocardia spp.* occurs mainly in immunocompromised patients, and it is usually considered in the differential diagnosis when an immunosuppressed patient presents with pulmonary, brain, or skin lesions. The clinical manifestations of *Nocardia* infections are very heterogeneous and nonspecific. Our patient had an unusual site of involvement. Since we were unable to review the initial pathology slides of the surgery performed in India, we do not know whether the initial mediastinal cystic masses were malignant thymic tissue or a manifestation of *Nocardia *infection. Both hypotheses are plausible. He could also have acquired the infection nosocomially during his surgery in India. Our patient had two predisposing factors for *Nocardia *infection: possible T-cell dysfunction from malignant thymoma and corticosteroid use. There is no clear or proven causal relation between *Nocardia* infection and myasthenia gravis or thymoma. Infection with *Nocardia asteroides* after corticosteroids therapy for malignant thymoma has been described in one report in 1993 [1]. To the best of our knowledge, this constitutes the second case ascribed to this species. The mediastinal and pericardial involvement is unusual but has been previously reported with some *Nocardia* species. For example, *Nocardia spp*. has been reported to cause purulent pericarditis [2, 3], cardiac tamponade [4], anterior mediastinal mass [5, 6], and mimicking pancoast tumor [7]. These reports of fatal involvement of the pericardium emphasize the necessity of early surgical intervention.

Concerning *N. asiatica*, 5 cases were reported in 2004, 2 from patients in Japan and 3 from Thailand [8] and have been described to cause brain abscesses, skin infection, and pulmonary infections [9–12]. This is the third report about *N. asiatica* from outside Japan and Thailand. The first report originated from Italy [11] and the second from Belgium [12].

As far as *Nocardia* treatment is concerned, there are no specific guidelines as the infection with such species is rare, and the treatment options were not entertained in clinical trials. Most of the treatment recommendations are based on animal models, basic research, case reports, and expert opinions. Treatment of disseminated *Nocardia *is usually with three antibiotics including imipenem or ceftriaxone, TMP-SMX, and amikacin for at least one year for nervous system involvement and according to clinical and radiological improvements [13].

Speciation and susceptibility testing of the isolated *Nocardia spp. *is essential to tailor the treatment regimen, as resistance is on the rise. In a study done in USA between 1995–2004, 765 *Nocardia *isolates were tested and 42% were resistant to TMP-SMX. Linezolid was the only antimicrobial agent for which 100% (269/269) of the tested isolates were susceptible [14]. A Belgian report described 100% susceptibility of *Nocardia spp.* to TMP-SMX, amikacin and linezolid [15]. Both of these two reports showed variable susceptibility to the other antimicrobial agents. A study from Kuwait showed that 93% of the *N. asteroides* isolates were resistant to sulfamethoxazole alone or in combination with trimethoprim [16]. Combination therapy is the standard of care for disseminated *Nocardia* infections. However, one report describes successful monotherapy with linezolid for disseminated *N. farcinica *in a heart transplant patient [17]. Linezolid has also been used in combination therapy with TMP-SMX [18].

## 3. Conclusion

*Nocardia spp.* infection has a heterogenous spectrum of manifestations and should be included in the differential diagnosis of an infectious etiology especially in patients with predisposing risk factors, such as, T-cell dysfunction, transplantation, hematological malignancies, steroids intake, infection with HIV, and cytotoxic therapy. Relation with myasthenia gravis or thymoma remains to be proven. Proper identification and susceptibility patterns must be determined due to increasing resistance to decide on optimal treatment in addition to surgical evacuation of collections.

## References

[1] Borges AA, Krasnow SH, Wadleigh RG, Cohen MH (1993). Nocardiosis after corticosteroid therapy for malignant thymoma. *Cancer*.

[2] Tabrizi SJ, Scott J, Pusey CD (1994). *Nocardia* pericarditis: a rare opportunistic infection. *British Medical Journal*.

[3] Hornick P, Harris P, Smith P (1995). *Nocardia asteroides* purulent pericarditis. *European Journal of Cardio-Thoracic Surgery*.

[4] Roubille F, Maxant G, Serre I (2010). Fatal systemic *Nocardia* infection revealed by cardiac tamponade. *Internal Medicine*.

[5] Kroe DM, Shulman N, Kirsch CM, Wehner JH (1997). An anterior mediastinal mass with draining sternal sinus tracts due to *Nocardia braziliensis*. *Western Journal of Medicine*.

[6] Jastrzembski SA, Teirstein AS, Herman SD, DePalo LR, Lento PA (2002). Nocardiosis presenting as an anterior mediastinal mass in a patient with sarcoidosis. *Mount Sinai Journal of Medicine*.

[7] Larobina M, McLean C, Davis BB (2004). Clinical-pathologic conference in general thoracic surgery: disseminated nocardiosis presenting as Pancoast syndrome. *Journal of Thoracic and Cardiovascular Surgery*.

[8] Kageyama A, Poonwan N, Yazawa K, Mikami Y, Nishimura K (2004). *Nocardia asiatica* sp. nov., isolated from patients with nocardiosis in Japan and clinical specimens from Thailand. *International Journal of Systematic and Evolutionary Microbiology*.

[9] Wakui D, Ito H, Ikeda R (2008). A complicated case of *Nocardia* brain abscess for differential diagnosis. *Neurological Surgery*.

[10] Matsumoto T, Shimizu T, Aoshima Y (2011). Endobronchial hamartoma with obstructive pneumonia due to *Nocardia asiatica*. *General Thoracic and Cardiovascular Surgery*.

[11] Iona E, Giannoni F, Brunori L, De Gennaro M, Mattei R, Fattorini L (2007). Isolation of *Nocardia asiatica* from cutaneous ulcers of a human immunodeficiency virus-infected patient in Italy. *Journal of Clinical Microbiology*.

[12] Verfaillie L, De Regt J, De Bel A, Vincken W (2010). *Nocardia asiatica* visiting Belgium: Nocardiosis in a immunocompetent patient. *Acta Clinica Belgica*.

[13] Ambrosioni J, Lew D, Garbino J (2010). Nocardiosis: Updated clinical review and experience at a tertiary center. *Infection*.

[14] Uhde KB, Pathak S, McCullum I (2010). Antimicrobial-resistant *Nocardia* isolates, United States, 1995–2004. *Clinical Infectious Diseases*.

[15] Glupczynski Y, Berhin C, Janssens M, Wauters G (2006). Determination of antimicrobial susceptibility patterns of *Nocardia* spp. from clinical specimens by Etest. *Clinical Microbiology and Infection*.

[16] Khan ZU, Al-Sayer H, Das Chugh T, Chandy R, Provost F, Boiron P (2000). Antimicrobial susceptibility profile of soil isolates of *Nocardia* asteroides from Kuwait. *Clinical Microbiology and Infection*.

[17] Rivero A, García-Lázaro M, Pérez-Camacho I (2008). Successful long-term treatment with linezolid for disseminated infection with multiresistant *Nocardia farcinica*. *Infection*.

[18] Shen Q, Zhou H, Li H, Zhou J (2011). Linezolid combined with trimethoprim-sulfamethoxazole therapy for the treatment of disseminated nocardiosis. *Journal of Medical Microbiology*.

